# The JAK1/STAT3/SOCS3 axis in bone development, physiology, and pathology

**DOI:** 10.1038/s12276-020-0445-6

**Published:** 2020-08-13

**Authors:** Natalie A. Sims

**Affiliations:** grid.1008.9 0000 0001 2179 088X St. Vincent’s Institute of Medical Research, and Department of Medicine at St. Vincent’s Hospital, The University of Melbourne, Parkville, VIC Australia

**Keywords:** Growth factor signalling, Metabolic bone disease

## Abstract

Bone growth and the maintenance of bone structure are controlled by multiple endocrine and paracrine factors, including cytokines expressed locally within the bone microenvironment and those that are elevated, both locally and systemically, under inflammatory conditions. This review focuses on those bone-active cytokines that initiate JAK–STAT signaling, and outlines the discoveries made from studying skeletal defects caused by induced or spontaneous modifications in this pathway. Specifically, this review describes defects in JAK1, STAT3, and SOCS3 signaling in mouse models and in humans, including mutations designed to modify these pathways downstream of the gp130 coreceptor. It is shown that osteoclast formation is generally stimulated indirectly by these pathways through JAK1 and STAT3 actions in inflammatory and other accessory cells, including osteoblasts. In addition, in bone remodeling, osteoblast differentiation is increased secondary to stimulated osteoclast formation through an IL-6-dependent pathway. In growth plate chondrocytes, STAT3 signaling promotes the normal differentiation process that leads to bone lengthening. Within the osteoblast lineage, STAT3 signaling promotes bone formation in normal physiology and in response to mechanical loading through direct signaling in osteocytes. This activity, particularly that of the IL-6/gp130 family of cytokines, must be suppressed by SOCS3 for the normal formation of cortical bone.

## Bone: signaling proteins in health and disease

Maintaining normal bone structure and strength depends on a group of signaling proteins called cytokines that bind to receptor molecules on cell surfaces. Natalie Sims at St. Vincent’s Institute of Medical Research and The University of Melbourne in Australia reviews the role of cytokines in a specific signaling pathway in bone development and disease. Two of the proteins in this pathway respond to cytokine activity, whereas the third inhibits the cytokines’ effects. Studies in mice and humans have identified links between specific bone defects and spontaneous or experimentally induced mutations in the genes that code for the three proteins. The review covers the significance of recent findings to several types of cells that form new bone, degrade bone as part of normal bone turnover, and sustain the structure of bone and cartilage.

## Introduction

Bone structure and strength are controlled by multiple pathways that regulate the function of cells within the skeleton, including the cells that form the bone matrix itself (osteoblasts), the cells that resorb bone either under normal or inflammatory conditions (osteoclasts), and the regulatory network of cells that reside within the bone matrix (osteocytes). In addition, bone size, particularly bone length, is determined by the activity of chondrocytes during bone development and growth. Osteoblasts, osteocytes, osteoclasts, and chondrocytes each respond to multiple cytokines during normal bone development and growth and under pathological conditions, particularly inflammation. Bone-active cytokines include those that signal through the JAK (Janus kinase) and STAT (signal transducer and activator of transcription) proteins and that are inhibited by the SOCS (suppressor of cytokine signaling) family of intracellular negative feedback proteins. There are >50 cytokines involved in diverse and complex JAK/STAT signaling pathways^1^. This review focuses specifically on the effects of skeletal JAK1, STAT3, and SOCS3, which transduce signals initiated by the IL-6 family cytokines that stimulate bone-forming cells (osteoblasts), bone-destroying cells (osteoclasts), and cartilage cells (chondrocytes). It also focuses particularly on the intracellular actions of these cytokines in osteoblasts, osteoclasts, osteocytes, and chondrocytes and their outcomes on bone structure.

## Introduction to JAK1/STAT3 signaling in bone

**Fig. 1:**
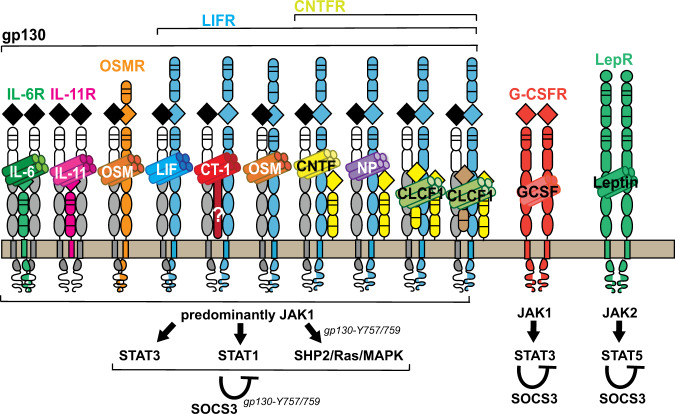
Bone-active cytokines and receptor complexes that depend on SOCS3-negative feedback. From left to right: a large number of cytokines form complexes with gp130. IL-6 and IL-11 bind to ligand-specific β-receptor subunits (IL-6R and IL-11R, respectively) to form complexes with glycoprotein 130 (gp130) homodimers. Oncostatin M (OSM) binds to its specific receptor (OSMR), which then recruits gp130 to form a heterodimer. The LIF (leukemia inhibitor factor) receptor is used by multiple cytokines. LIF itself signals through a complex containing the ligand bound to a heterodimer of LIF receptor (LIFR) and gp130. Cardiotrophin 1 (CT-1) also transduces signals through LIFR and gp130 and, potentially, a CT-1-specific receptor subunit that remains undefined. In addition to its ability to transduce signal through OSMR, OSM is also capable of signaling through a gp130:LIFR heterodimer. A subfamily of cytokines transduce signals through a complex containing gp130:LIFR and the ciliary neurotrophic factor (CNTF) receptor (CNTFR). The simplest complexes are formed by CNTF and neuropoietin (NP), but additional components are required for CLCF1 (Cardiotrophin-like cytokine factor 1) signaling. CLCF1 is secreted as a complex bound to either a soluble form of CNTFR or to cytokine receptor-like factor (CRLF). All receptors that bind gp130 activate predominantly JAK1 (although there are some that can bind JAK2 and TYK2), and once JAK1 is phosphorylated, STAT3, STAT1, and SHP2/Ras/MAPK signaling is activated. SHP2/Ras/MAPK signaling is mediated through tyrosine 757 (mouse) or 759 (human) in gp130. Each of these pathways is subject to negative feedback via SOCS3, which also depends on tyrosine 757/759. Two additional bone-active cytokines depend on SOCS3: granulocyte colony stimulating factor (G-CSF), which acts through a G-CSF receptor (G-CSFR) homodimer followed predominantly by JAK1 and STAT3 signaling, with negative feedback through SOCS3 and leptin, which acts through a leptin receptor (LepR) homodimer, followed by JAK2 and STAT5 signaling, which appears to also be suppressed by SOCS3.

JAK/STAT cytokines transduce signals by binding to specific receptor complexes on the target cell surface^1^; each receptor complex includes a signaling receptor subunit that contains an intracellular domain constitutively associated with an inactive Janus kinase (JAK) protein (Fig. 1). Upon formation of the ligand–receptor complex, the JAK protein associated with the receptor is activated by transphosphorylation. JAK activation triggers the phosphorylation of tyrosine on a cytoplasmic tail subunit of the receptor at docking sites for STAT proteins. This phosphorylation leads to STAT protein phosphorylation and dimerization and dimer translocation to the nucleus, where they bind DNA and activate the transcription of responsive genes that influence cell behavior. Another set of gene products transcribed in response to STAT signaling is the suppressors of cytokine signaling (SOCS) proteins, which provide negative feedback to the receptor and prevent continuous signaling by switching off the signaling cascade.

There are multiple JAK, STAT, and SOCS proteins, each activated by specific cytokine–receptor complexes^1^. This review discusses JAK1 and STAT3 signaling and their requirement for SOCS3-negative feedback within bone because it is a pathway stimulated by IL-6 family cytokines, which bind the gp130 (glycoprotein 130) IL-6 receptor subunit and are essential for the normal skeletal development of mice and humans^2–4^.

The IL-6 family of cytokines is extensive and includes IL-6, IL-11, oncostatin M (OSM), cardiotrophin 1 (CT-1), leukemia inhibitory factor (LIF), ciliary neurotrophic factor (CNTF), and a number of other complex cytokines that transduce signals through the CNTF receptor. Each of these cytokines regulates bone mass, either in homeostasis or in inflammatory pathologies^5,6^.

In general terms, many IL-6 family cytokines act within the osteoblast lineage to stimulate bone formation, an essential function for normal bone structure^7–11^. This bone formation effect is, at least in the case of oncostatin M, mediated primarily through STAT3 signaling^12^. STAT3 signaling induces osteoblast differentiation by stimulating the expression of transcription factors that induce osteoblast differentiation, such as C/EBPδ and C/EBPβ^9,10^. These STAT3-dependent cytokines also suppress gene products that inhibit osteoblast differentiation, such as sclerostin^10^ and ZFP467^13^. STAT3 signaling, at least in the case of oncostatin M, also induces matrix metalloproteinase 13 (MMP13)^12^, which modifies osteocyte behavior within the bone matrix^14^. These actions of IL-6 family cytokines are required for normal levels of bone formation in physiology but also induce pathological bone formation, such as heterotopic ossifications after spinal cord injury^15^.

IL-6 family cytokines also stimulate osteoclast formation through indirect actions on osteoblasts^16^. They induce STAT3 signaling in the osteoblast lineage to stimulate the transcription of a range of factors that trigger osteoclast precursors to initiate osteoclast differentiation and bone resorption, such as RANKL^17^ and the chemotactic factor CXCL1^12,18^. This induction of osteoclast formation is critical for the bone loss associated with inflammatory conditions such as rheumatoid arthritis, colitis, or estrogen deficiency^5,6,19–21^ owing to osteoclast-mediated bone resorption. IL-6 family cytokines are also required for normal osteoclast formation during endochondral ossification in the neonatal skeleton and for the related process at the growth plate during longitudinal growth when cartilage is destroyed prior to its replacement with bone^2,9,11,22^. This review focuses on intracellular signaling pathways activated by these cytokines and the contexts in which they are important to the skeleton.

The JAK1/STAT3/SOCS3 pathway is also activated by G-CSF^1^, which inhibits bone formation and promotes osteoclast formation^23,24^ through indirect actions mediated by macrophages^23,25^. The leptin receptor is also subject to negative feedback signaling from SOCS3, but as it transduces signals through JAK2 and STAT5^1^ and its direct actions in bone remain highly controversial^26^, leptin signaling pathways are not discussed in this review.

## Effects of JAK1 inhibition or activation on bone

Many signaling cytokines depend on JAK1, and therefore, perhaps unsurprisingly, JAK1-null mice generated by two different laboratories died during early embryogenesis^27,28^. JAK1 can be activated by IL-6 family cytokines and G-CSF, and it is a signal mediator for class II cytokine receptor cytokines (interferon-α/β interferon γ, and IL-10) and γ_c_ receptor cytokines (IL-2, IL-4, IL-7, IL-9, and IL-15)^27,29^ and activates multiple STAT proteins in addition to STAT3^29^. Genetically altered mouse models and JAK inhibitor treatment studies have revealed that JAK1 activation is critical for increased osteoclast formation under inflammatory conditions (Table 1).

**Table 1:** Phenotypes of mouse models with modified JAK1 signaling.

Mouse model	Targeting?	Genes modified	Phenotype	References
*Jak1*^*−/−*^	Germline	JAK1 deletion	Embryonic lethal, stunted embryos	^27^
*MMTV‐Cre.Jak1*^*fl/fl*^	Germline	JAK1 deletion	Embryonic lethal, stunted embryos	^28^
Tofacitinib treatment	Systemic	JAK1/3 inhibition	Protected against localized bone resorption caused by inflammation. Possible systemic protection against elevated resorption caused by inflammation.	^31–33^
Ruxolitinib treatment	Systemic	JAK1/2 inhibition	Protected against age-related bone resorption.	^34^
*Jak1*^*S645P+/−*^	Germline	JAK1 activation	Adult trabecular and cortical bone mass levels were both low due to elevated bone formation and resorption, indicating secondary systemic lupus erythematosus-like symptoms.	^48^

Selective JAK1 inhibitors, such as tofacitinib and baricitinib, are used to treat rheumatoid arthritis (reviewed in ref. ^30^). In mouse and rat models of rheumatoid arthritis, tofacitinib (a selective JAK1/3 inhibitor) administered at initial symptom onset blocked further inflammation and subsequent local joint destruction (including that caused by both bone erosion and cartilage damage)^31–33^ and reduced RANKL expression in arthritic rat joints^32^. Although secondary osteoporosis occurs in rheumatoid arthritis, tofacitinib showed no systemic protective effects on bone mass or strength in these studies^33^. However, tofacitinib prevented the reduction in bone hardness associated with arthritis in cortical and trabecular bone at noninflamed sites, suggesting that tofacitinib inhibits bone remodeling, a finding that was confirmed by the reduction in serum markers of bone formation and resorption in arthritic animals^33^. In summary, tofacitinib may both reduce focal damage and have a limited protective effect against secondary osteoporosis in rheumatoid arthritis. Whether this action only results from an anti-inflammatory effect is not known, as nonarthritic animals were not treated.

There is also some evidence that JAK1/2 inhibition may suppress age-related bone loss by inhibiting osteoclastogenesis, as indicated by 22-week-old C57BL/6 mice treated with ruxolitinib showing greater bone mass and strength with reduced osteoclast numbers compared with controls, but no change in osteoblast numbers^34^. Under normal conditions, a low number of osteoclasts leads to reduced production of osteoclast-derived coupling factors, which are agents that promote osteoblast differentiation; this would lead to reduced osteoblast numbers^35^. The finding of normal osteoblast numbers in the presence of low numbers of osteoclasts suggests that osteoclast-to-osteoblast coupling may be JAK1/2 dependent. The authors ascribed the effect of ruxolitinib on these cells to its anti-senescence effect. However, the finding is also consistent with an earlier model showing that IL-6-dependent pathways mediate coupling in bone with a high degree of remodeling^36^ and that ovariectomy-induced bone loss may, at least in part, depend on IL-6 signaling^19^ and T cell activation^37^.

A very recent study reported a protective effect of high-dose tofacitinib on bone mass in young mice, a finding consistent with the anti-osteoclastogenic effects described above. The authors suggested that tofacitinib may have an “osteoanabolic” effect—this implies an effect that increases bone formation and increases bone mass^38^. Unfortunately, bone formation was not assessed in vivo in this study. In addition, the protective effect of tofacitinib was only studied in young mice^38^ during a period when trabecular bone loss usually occurs owing to normal remodeling of the prepubertal trabecular structure^39^. These findings confirm that tofacitinib may protect against bone loss, likely by inhibiting osteoclast formation, as previously reported. Whether it is truly anabolic remains to be clarified.

JAK1 inhibitors impair osteoclast formation through an indirect mechanism rather than by blocking signals within the osteoclast lineage. As observed in inflamed tissue, these agents change the ability of accessory cells to support osteoclast formation. For example, tofacitinib and the selective JAK1/2 inhibitor baricitinib reduce the ability of osteoblasts to support osteoclast formation but do not directly inhibit osteoclastogenesis^31,40^. The same was observed with shRNAs directed to either JAK1 or JAK2^40^. The requirement for osteoblast support for JAK-mediated signaling is consistent with the known action of many cytokines, including IL-6 family cytokines, to stimulate osteoclast formation by acting on supportive osteoblast lineage stromal cells rather than by directly acting on osteoclast precursors^16^. These cytokines all stimulate osteoclast formation by promoting the expression of RANKL, an osteoclastogenic factor, by accessory cells, including osteoblast lineage cells^41,42^ and T cells, during inflammation^43^.

Indeed, baricitinib reduced RANKL expression in cultured osteoblasts in response to osteoclastogenic stimuli^40^, and tofacitinib reduced RANKL production in cultured T cells in a dose-dependent manner^32^. Tofacitinib and baracitinib inhibited osteoclast formation both when IL-6 family cytokines were used as a stimulus, and when osteoclast formation was induced by prostaglandin E2 or a combination of 1,25-dihydroxyvitamin D3 and prostaglandin E2^31,40^. These findings imply that multiple osteoclastogenic stimuli that do not directly transduce JAK1 signaling depend on it to stimulate osteoclast formation; this supposition is consistent with the results of early studies showing that osteoclast formation stimulated by IL-1, PTH, 1,25-dihydroxyvitamin-D3 and prostaglandin E2 was reduced when a gp130-neutralizing antibody was added to cultures^44^. Using a cytokine array, the most recent work showed that 1,25-dihydroxyvitamin D3 and prostaglandin E2 treatment may stimulate JAK1 indirectly, by increasing the protein levels of IL-11, IL-6,and LIF in the culture system, presumably through secretion by osteoblasts^40^.

The direct effects of JAK1 inhibitors on bone formation or on the functions of osteocytes in vitro have not been reported, even though IL-6 family cytokines promote bone formation by direct action on these cells^7–11^. A single in vitro study used high-throughput screening of human mesenchymal stem cells and found that tofacitinib, baricitinib, and ruxolitinib (a JAK1/2 inhibitor) inhibited alkaline phosphatase activity in these cells, but follow up in vivo studies were limited to a poorly described ectopic ossification model, and only ruxolitinib was used^45^. Although suggestive of a direct effect on bone formation, there is clearly much more to be learned with respect to JAK1 inhibitors.

Similarly, little is known about the effects of direct JAK1 inhibition on chondrocytes. Given that IL-6 family cytokines have been implicated in osteoarthritis^46^ and that gp130 inactivation may suppress osteoarthritis-induced cartilage damage^47^, further work into JAK1 inhibition with respect to osteoarthritis is warranted.

Does increased JAK1 activation cause bone loss? The data described above using JAK1 inhibitors certainly suggest that bone resorption in inflammatory arthritis is caused by JAK1 activation. In addition, in a mouse model with a germline-activating mutation in JAK1, the mature adult trabecular and cortical bone mass levels were low^48^. This finding was associated with high levels of bone formation and resorption markers in the serum and increased endocortical circumference (suggesting cortical thinning). Hypophosphatemia was observed, suggesting the development of osteomalacia, but kidney calcium and phosphate levels appeared normal. These findings seemed to model systemic lupus erythematosus (SLE). Certainly, the low bone mass is consistent with well-known skeletal fragility of SLE patients owing to the detrimental effects of inflammation on the skeleton^49^.

Overall, the current data suggest that direct activation of JAK1 signaling in osteoblasts may promote their differentiation, whereas increased osteoclast formation owing to JAK1 activation is secondary to the effects of inflammatory T cells, not owing to direct effects on the osteoclast lineage (Table 1).

## Effects of STAT3 inhibition on bone

As observed with JAK1 deletion, STAT3 germline deletion is lethal to early embryos^50^, reinforcing the importance of this pathway in multiple biological functions (Table 2). Since the skeleton had not yet formed in these embryos, this mutant mouse could not provide any information about the role(s) of STAT3 in skeletal formation. This very early lethality differs from mice with germline gp130 inactivation, which were initially shown to die before E13.5, but when backcrossed to a different strain, they survived until the perinatal period^3^. Mice with G-CSFR or leptin receptor deletion survived to adulthood^51,52^. This highlights that multiple receptor pathways converge on STAT3 signaling and that loss of all three pathways is inconsistent with survival.

**Table 2:** Phenotypes of mouse models and human conditions with modified STAT3 signaling.

Model	Effect on STAT3	Phenotype	References
*Stat3*^*−/−*^	STAT3 deletion in all cells	Early embryonic death (day E8.5).	^50^
Hyperimmunoglobulinemia E (hyper-IgE) syndrome (human)	STAT3-DNA binding reduced in all cells	Low bone mineral density, recurrent fractures, craniofacial abnormalities.	^58^
SA/SA and SA/ −	Reduced STAT3 phosphorylation in all cells	SA/SA phenotypically normal; lower STAT3 phosphorylation in SA/−, associated with 75% perinatal lethality and reduced skeletal size.	^54^
*Dmp1Cre.Stat3*^*fl/fl*^	STAT3 deletion in osteocytes	Low bone mass owing to impaired bone formation; reduced bone formation response to mechanical loading.	^73^
*Col1*α1(*2.3* *kb) Cre; Stat3*^*flox*/flox^	STAT3 deletion in osteoblasts and osteocytes	Low trabecular bone mass owing to reduced bone formation; normal bone length; reduced bone formation response to mechanical loading.	^68,76^
*Col1*α1(*3.6* *kb) Cre; Stat3*^*flox/flox*^	STAT3 deletion in chondrocytes, osteoblasts, and osteocytes	Very small skeletal size; low trabecular bone mass owing to reduced bone formation and increased osteoclast formation.	^76^
*Prrx1Cre; Stat3*^*flox*/flox^	STAT3 deletion in chondrocytes, osteoblasts, and osteocytes	Reduced skeletal size and postnatal limb curvature; no data on bone mass.	^55^
*TCre.Stat3*^*f/f*^	STAT3 deletion in mesoderm-derived cells	Shortened limbs at birth and limb curvature in postnatal development; no data on bone mass.	^55^
*Tie2(Tek)Cre.Stat3*^*f/f*^	STAT3 deletion in endothelial and hematopoietic cells	Reduced skeletal size and bone mass owing to impaired bone formation and increased resorption; secondary to inflammatory colitis.	^81^
*Socs3*^*−/−*^	SOCS3 deletion; elevated STAT3 signaling in all cells	Embryonic lethality.	^84,85^
*VavCre.Socs3*^*f/f*^	Elevated STAT3 signaling in endothelial and hematopoietic cells	Joint inflammation, low bone mass owing to increased osteoclast formation both in joints and systemically; increased osteoblast formation.	^86^
*Dmp1Cre.Socs3*^*f/f*^	Elevated STAT3 signaling in osteocytes	Increased cortical porosity owing to delayed development of cortical bone, particularly in females; elevated bone formation and increased resorption later in life.	^87^
*Dmp1Cre.Socs3*^*f/f*^*.IL-6*^*−/−*^	Elevated STAT3 signaling in osteocytes but not downstream of IL-6	Increased cortical porosity owing to delayed development of cortical bone in males and females.	^87^
*Dmp1Cre.Socs3*^*f/f*^*.Il6st* ^*f/f*^	Elevated STAT3 signaling in osteocytes but not downstream of gp130	Rescue of the *Dmp1Cre.Socs3*^*f/f*^ phenotype.	^91^
*Col2Cre.Socs3*^*f/f*^	Elevated STAT3 signaling in chondrocytes, osteoblasts and osteocytes	Increased cortical porosity, and reduced bone size.	^89^

STAT3 is phosphorylated at tyrosine 705 by JAK1, and its activation is optimized when it is also phosphorylated at a second site, serine 727^53^. When an inactivating point mutation was introduced at serine 727 (SA mutation), the mice were viable and grossly normal^54^, indicating that this pathway is dispensable for homeostasis. When these mice were crossed with STAT3 heterozygous mice, which are viable and grossly normal, STAT3 reporter activity (a measure of transactivation) was reduced compared with that in STAT3^+/−^ mice, thus reducing STAT3 activation without early embryonic lethality^54^. These mice (termed SA/−) survived birth, but 75% died before reaching 3 weeks of age. The SA/− mice that survived had impaired growth, which developed in late fetal development, and despite some catch-up growth, these mice did not fully recover; this outcome was attributed to reduced circulating IGF-I levels^54^. The skeletal phenotype was not specifically examined, but the reduced body size may also reflect an intrinsic modification to JAK/STAT or SHP2 signaling in chondrocytes. This explanation is consistent with findings in *Prrx1Cre*-targeted STAT3-deficient mice, as outlined below^55^.

Patients with autosomal dominant hyperimmunoglobulinemia E (hyper-IgE) syndrome (HIES)/Job syndrome exhibit mutations in STAT3 that reduce its ability to bind DNA^56,57^. This rare multifactorial condition includes low bone mineral density, recurrent fractures, and craniofacial and skeletal abnormalities^58^. Whether the bone defect primarily reflects STAT3 loss in bone cells or is secondary to effects on other organs is unclear, partly because the cellular defects critical for the bone abnormalities remain undefined. The osteopenia in these patients has been associated with increased osteoclast activity^59^ based on an early study showing that osteoclast precursors from these patients have increased resorptive ability in vitro^60^. However, the craniofacial defects suggest additional defects in osteoblast activity; owing to the rarity of the condition, mechanistic studies have been challenging.

## Investigations of STAT3 and SHP2 signaling downstream of gp130 in bone

More information on the specific roles of STAT3 signaling in comparison with other intracellular pathways has been collected from studies of mice with mutations targeting specific downstream pathways of the coreceptor gp130 subunit, which binds IL-6 cytokine family ligands (Table 3). In addition to initiating signaling through STAT3, gp130 also induces STAT1 and SHP2/Ras/Erk/MAPK intracellular pathways. Gp130 is essential for normal skeletal development; gp130-null mice exhibited short, misshaped bones with many osteoclasts destroying the newly formed bone and very few osteoblasts^2^. This early lethal phenotype of bone development is also observed in humans with mutations that inactivate gp130^4^, highlighting its critical role in bone development.

**Table 3:** Phenotypes of mouse models and human conditions with modified gp130 signaling.

Model	Cells targeted	Effect on signaling	Phenotype	References
*gp130*^*−/−*^	Germline	No gp130 signal	Embryonic lethality or perinatal lethality (depending on background strain); short, misshapen bones; low bone mass; increased osteoclasts at the growth plate.	^2,3^
Stüve-Wiedemann syndrome (extended)	Human mutation	Varying degrees of gp130 inactivation	Varying degrees of lethality; short, misshapen bones; increased osteoclasts at the growth plate.	^4^
*gp130D/D*	Germline	No gp130 signal	Perinatal death; normal body size; skeletal phenotype not specifically assessed.	^61^
gp130-DN	Overexpression of truncated soluble gp130	gp130 suppression	No skeletal analysis.	^64^
PEPCK-sgp130-Fc	Secretion of a soluble gp130 homodimer into the bloodstream	Suppressed IL-6 *trans-*signaling at low levels	No skeletal phenotype at low levels of sgp130-Fc; low bone mass, reduced bone size, and impaired bone strength at high levels of sgp130-Fc.	^8^
*gp130*^*FXXQ/FXXQ*^,	Germline knock-in of a mutated human receptor	gp130 hyperactivates SHP2/Ras/MAPK and does not activate STAT1 or STAT3	Neonatal death owing to suckling defect; normal size; no skeletal analysis.	^61^
*gp130*^*ΔSTAT/ΔSTAT*^	Germline knock-in of a mutated murine receptor	gp130 hyperactivates SHP2/Ras/MAPK and does not activate STAT1 or STAT3	Inflammatory joint disease, reduced bone size owing to defective chondrocytes and early growth plate closure.	^36,66^
*gp130*^*Y757F/Y757F*^	Germline knock-in of a mutated murine receptor	gp130 hyperactivates STAT1/STAT3 and does not activate SHP2/Ras/MAPK	Low bone mass with elevated bone resorption and bone formation.	^36^
*gp130*^*Y757F/Y757F*^.IL-6^*−/−*^	Germline knock-in of the mutated murine receptor crossed with germline IL-6 knockout	gp130 (downstream of all cytokines other than IL-6) hyperactivates STAT1 and STAT3 and does not activate SHP2/Ras/MAPK	Low bone mass associated only with elevated bone resorption.	^36^
*gp130*^*Y757F/Y5757F*^*.Stat1*^*−/−*^	Germline knock-in of mutated murine receptor and deletion of STAT1	gp130 hyperactivates STAT3 and does not activate SHP2/Ras/MAPK or STAT1	Normal bone mass. Rescue of *gp130*^*Y757F/Y5757F*^ phenotype.	^12^
*gp130*^*Y757F/Y5757F*^*.Stat3*^*+/−*^	Germline knock-in of the mutated murine receptor and hemizygous STAT3 deletion	gp130 hyperactivates STAT1 and does not activate SHP2/Ras/MAPK or STAT3	Low bone mass associated with elevated bone resorption and bone formation.	^12^
*gp130*^*F759/F759*^	Germline knock-in of the mutated human receptor	gp130 hyperactivates STAT1 and STAT3 and does not activate SHP2/Ras/MAPK	Increased trabecular bone volume owing to increased bone formation.	^68^
*Osx1Cre.gp130*^*f/f*^	Osteoblast and osteocyte-targeted deletion of the gp130 transmembrane domain	Reduced gp130 signaling in osteoblasts and osteocytes	Reduced trabecular bone volume owing to reduced bone formation; increased cortical bone growth.	^7^
*Dmp1Cre.gp130*^*f/f*^	Osteocyte-targeted deletion of the gp130 transmembrane domain	Reduced gp130 signaling in osteoblasts and osteocytes	Reduced trabecular bone volume owing to reduced bone formation; increased cortical bone growth.	^7,75^
*Dmp1Cre.Il6st*^*f/f*^	Osteocyte-targeted deletion of gp130	Reduced gp130 in osteoblasts and osteocytes	Reduced trabecular bone volume owing to reduced bone formation; increased cortical bone growth.	^91^
*CtskCre.gp130*^*f/f*^	Osteoclast-targeted deletion of the gp130 transmembrane domain	Reduced gp130 signaling in osteoclasts	Reduced trabecular bone volume owing to reduced bone formation; reduced cortical bone growth.	^92^

Another model of gp130 deletion is the gp130D/D mouse, which lacks both the transmembrane and the intracellular signaling domains of gp130. This mouse is incapable of signaling by any of the cytokines that lead to gp130 homodimer formation (e.g., IL-6 and IL-11, see Fig. 1). The gp130D/D mice, like gp130-null mice, died during the perinatal period^61^. In contrast to gp130-null mice, however, body size of the gp130D/D mice was normal, and bone size was not obviously affected (although it was not specifically assessed)^61^. Normal bone size in these mice would indicate that the cytokines that bind gp130 and form heterodimers with other signaling receptors (such as LIF and OSM, see Fig. 1) retain some ability to transduce signals through an extant gp130 extracellular domain, even in the absence of its intracellular domain. Such a conclusion is consistent with early lethality, defective bone length in mice with LIFR deletion^22^, and normal bone length in mice with IL-6 or IL-11R deleted^62,63^.

Transgenic gp130 overexpression has been used to suppress intracellular gp130 responses in vivo. In an early mouse model, gp130 signaling was inhibited by the transgenic expression of a dominant negative membrane-bound gp130; this inhibited the phosphorylation of both gp130 and STAT3 downstream of gp130. The inhibition of gp130 led to impaired thymocyte and splenocyte development, but unfortunately, no skeletal analysis was performed in this study^64^. More recently, mice were designed to express a synthetic gp130 extracellular domain homodimer (sgp130-Fc) in their bloodstream^65^. This synthetic homodimer was designed to inhibit IL-6 *trans-*signaling^21^. The classical understanding of IL-6 signaling is that the cytokine binds to its specific membrane-bound receptor, IL-6R, which binds to a gp130 homodimer (*cis-*signaling) to initiate JAK/STAT signaling. In *trans-*signaling, IL-6 induces signal transduction by binding to a soluble form of IL-6R and then initiates signaling through a gp130 homodimer. Soluble IL-6R may be produced locally or found in the circulation, where it is elevated in inflammatory conditions. When analyzed in adulthood, mice expressing sgp130-Fc had normal bone size, bone shape, and trabecular bone mass^8^, confirming that IL-6 *trans-*signaling had a key role in the skeleton that was restricted to inflammation- or ovariectomy-induced bone loss^19^. However, in the same mouse line, when circulating sgp130-Fc levels were very high, to an extent that they were likely to induce off-target effects on other IL-6 family cytokines, reduced bone size, low trabecular and cortical bone mass, and impaired bone strength were observed^8^. These results indicate that gp130 signaling in the postnatal skeleton is required for normal bone growth and strength, but whether it is required because of the inhibition of direct signaling in chondrocytes, osteoblasts, osteocytes, or osteoclasts or is secondary to its effects on other cell types, such as inflammatory cells, remains unknown.

Two independent laboratories designed models to test the absence of gp130-dependent STAT1 and 3 signaling, but inadvertently developed models of hyperactivated SHP2 signaling. First, a human gp130 was introduced with inactivating mutations in the four tyrosines (with YxxQ motifs) responsible for STAT3 phosphorylation (*gp130*^*FXXQ/FXXQ*^). In another model (*gp130*^*ΔSTAT/ΔSTAT*^), gp130-dependent STAT3 signaling was ablated by a point mutation in one STAT3 YxxQ tyrosine and deletion of the remaining three tyrosines was achieved by truncation^66^. In both models, STAT3 phosphorylation was blocked, but there was reciprocal upregulation of SHP2 phosphorylation^61,66^. The phenotypes were different. The *gp130*^*FXXQ/FXXQ*^ mice exhibited neonatal death because of a suckling defect, although their body size was normal, but their bones were not analyzed in the study^61^. In contrast, the *gp130*^*ΔSTAT/ΔSTAT*^ mice survived until adulthood, exhibited inflammatory joint disease with floating cartilage islands within some joints^66^ and shortened bones owing to early growth plate closure^36^. It remains unclear why the two models exhibited different survival and bone length phenotypes. As gp130-STAT3-dependent signaling was ablated, the difference likely reflects differences in the level of SHP2 activation in the two models, but they have not been compared directly.

In contrast, mice generated with SHP2/Ras/MAPK signaling pathway blocked downstream of gp130 exhibited upregulated and prolonged gp130-dependent STAT signaling owing to interference with the SOCS3-binding site on the receptor^61,67^. In these mice, gp130-dependent SHP2 phosphorylation was blocked by the introduction of point mutations, either at murine gp130 tyrosine 757 (*gp130*^*Y757F/Y757F*^)^67^ or at the equivalent in an introduced human gp130 tyrosine 759 (*gp130*^*F759/F759*^)^61^. Again, different phenotypes were observed. The *gp130*^*Y757F/Y757F*^ mice exhibited low trabecular bone mass, caused by a profound increase in osteoclast formation and bone resorption^36^. This increase in osteoclast formation was intrinsic to the monocyte/macrophage lineage^36^, indicating that gp130-dependent STAT signaling promotes osteoclastogenesis, even though no direct effect of gp130 cytokines on osteoclast differentiation has been identified. IL-6 family cytokines are generally understood to indirectly stimulate osteoclast formation by triggering supporting stromal cells^16^ to produce RANKL^41,42^. The elevation in osteoclast formation in the *gp130*^*Y757F/Y757F*^ mice was not observed in the alternate *gp130*^*F759/F759*^ model^68^ for reasons that remain unclear.

Both mouse models with hyperactivated STAT signaling downstream of gp130 (the *gp130*^*Y757F/Y757F*^ and *gp130*^*F759/F759*^ mice) exhibited increased osteoblasts^36,68^. Further work on the *gp130*^*Y757F/Y757F*^ mice indicated that this increase was not intrinsic to the osteoblast lineage and depended on IL-6, indicating that osteoblast formation is coupled to that of osteoclasts, at least in this instance, through an IL-6-dependent pathway that is essential for the normal control of bone remodeling^36^. The increased osteoblast and osteoclast numbers in the *gp130*^*Y757F/Y757F*^ mice were both recently confirmed to depend specifically on elevated STAT3 signaling^12^. When the mice were crossed with STAT1-knockout mice, the high osteoblast and osteoclast numbers were maintained. In contrast, when *gp130*^*Y757F/Y757F*^ mice were crossed with a STAT3 heterozygote, the phenotype was rescued; that is, both the osteoclast and osteoblast numbers were reduced^12^. This suggests that the SOCS3-mediated negative feedback to gp130 is required to restrain osteoclastogenesis throughout the osteoclast lineage.

## Effects of cell lineage-targeted STAT3 deletion on the skeleton

As the above mouse models and humans with gp130 and related receptor mutations have defective signaling in all cell types, researchers have not been able to discern whether the skeletal defects are a direct consequence of the mutation in osteoblasts, osteoclasts, or chondrocytes or are secondary to other defects, such as increased susceptibility to colitis^67^, joint inflammation^66^, or enhanced immune responses^61^. This has been resolved to some extent through cell-specific STAT3 deletion models (Table 2).

The osteoblast lineage both forms bone and provides support for osteoclast formation^69^. Osteoblasts are also precursors to osteocytes, an extensive network of interconnected cells embedded in the bone matrix^70^ that regulate bone formation and mineralization and may also mediate signals that support osteoclast formation^69,71^. Even though STAT3 signaling in the osteoblast lineage is required to support osteoclast formation in vitro^72^, studies in STAT3-deficient mice have revealed that its critical function in these cells in vivo is to promote bone formation in the trabecular network, and to limit periosteal growth.

STAT3 deletion in osteocytes with a *Dmp1Cre* construct suppressed bone formation and led to fewer osteoblasts in the adult trabecular network^73^. Despite the reduction in bone formation, there was no significant effect on trabecular bone mass; in fact, there was an increase in bone width^73^. These mice also exhibited an impaired response to mechanical loading, which normally induces bone formation^73^, a finding consistent with early reports showing that mechanical loads promote STAT3-dependent gene expression in vivo^74^. The low level of trabecular bone formation in these mice combined with their increased bone width is a phenocopy of *Dmp1Cre*-targeted gp130-knockout mice^7,75^, confirming that IL-6 family cytokines, not leptin or G-CSF, are the main STAT3-dependent cytokines that act through osteocytes to regulate physiological bone formation.

STAT3 deletion early in osteoblast differentiation (in committed osteoblasts and osteocytes) based on *Col1α1* (2.3 kb).*Cre*^68,76^ or the *Col1*α1 (3.6 kb)*.Cre*^76^ model, which is active earlier in the lineage, confirmed the role of STAT3 as an essential stimulus of osteoblast activity. In each of these models, bone formation, both at the baseline^68,76^ and in response to mechanical load^76^, was suppressed. Thus, these models are phenocopies of the more restrictive *Dmp1Cre-*targeted STAT3 null model, which confirms that STAT3 signaling regulates bone formation through osteocytes. These findings also indicate that, although IL-6 family cytokines can promote the differentiation of osteoblast precursors directly^77,78^, their action in osteocytes is most critical for normal bone physiology. These critical functions include their ability to suppress the expression of sclerostin^8,10^, an osteocyte-derived bone formation inhibitor, through a STAT3-dependent mechanism^12^.

Bone lengthening depends on precisely controlled proliferation and hypertrophy of growth plate chondrocytes^79^. Shortened bones were observed in neonatal mice lacking gp130^2^ or LIFR^22^ and in adult mice lacking LIF^11^, suggesting that STAT3 signaling regulates longitudinal bone growth. Whether this modulation reflects direct action on chondrocytes or is secondary to other defects is not known. When *Prrx1Cre* was used to target STAT3 deletion to osteoblast/chondrocyte progenitors, bones were shorter, but only after birth^55^. In contrast to global STAT3-knockout mice, *Prrx1Cre*-driven STAT3 null mice showed an extended hypertrophic zone even at birth, which also led to limb curvature. Direct and indirect effects of STAT3 on bone length were confirmed in the same study; mice with mesoderm-targeted deletion of STAT3 also exhibited shortened limbs at birth and developed postnatal bone curvature^55^. The extended chondrocyte hypertrophic zone reported in both models differed from the hypertrophic zone in the gp130-, LIFR-, and LIF-null mice, which each exhibited reduced growth plate width owing to increased osteoclast activity^2,11,22,63^. The phenotype of the *Prrx1Cre*-targeted STAT3 null mice suggests that the primary function of STAT3 in chondrocytes is to promote their progression to hypertrophy and thereby promote bone lengthening. The limb curvature that developed in these mice after they were born was similar to that of other, unrelated mice with defective growth plates^80^ and was likely secondary to a mechanical defect in the bone, as it was associated with an inability to properly support ambulation similar to defects observed in young individuals with rickets.

In the hematopoietic lineage, the use of Tie2*(Tek)Cre* to target STAT3 deletion in early hemopoietic and endothelial cells resulted in low trabecular bone mass and impaired skeletal growth^81^. This outcome was most likely the result of a secondary effect of inflammatory colitis^82^. Colitis has long been known to be associated with osteopenia (low bone mass), which is caused by both a high level of bone resorption by osteoclasts and a low level of bone formation by osteoblasts^83^. This association provides further evidence that STAT3 signaling controls osteoclastogenesis only under pathological conditions, particularly those associated with local or systemic inflammation.

## Effects of SOCS3 deficiency and elevated STAT3 activation

SOCS3 is a direct target of STAT3 signaling and provides negative feedback to STAT3 and, to a lesser extent, STAT1. SOCS3 is essential for life: the hyperactivation of STAT3 signaling that results from SOCS3 deletion leads to death during embryogenesis, with most mice dying prior to skeletogenesis at E13.5 owing to erythrocytosis and placental defects^84,85^. As for other studies, cell-specific knockouts have been essential for identifying the role of SOCS3 in bone biology and determining the consequences of cell-specific hyperactivation of this pathway (Table 2).

Prolonged STAT3 activation owing to targeted deletion of SOCS3 in hematopoietic and endothelial cells using *VavCre* led to spontaneous joint inflammation^86^. Osteoclast formation was increased in vitro and in vivo, both at the inflamed joints, and as a secondary consequence in the peripheral skeleton, possibly owing to elevated IL-6 production. Osteoblast formation was also increased as a secondary effect of increased osteoclast formation, and low trabecular bone mass was a result, as observed in the *gp130*^*Y575F/Y575F*^ mice^36^.

Given that bone formation was reduced in mice with osteocyte-specific deletion of gp130^7^, it was not particularly surprising that mice with *Dmp1Cre-*targeted deletion of SOCS3 exhibited a high level of bone formation^87^. What was surprising was the context- and sex-specific changes observed in these mice. During early bone growth, the trabecular bone mass was elevated in the *Dmp1Cre.Socs3*^*f/f*^ mice, but as the mice passed through puberty and the level of bone remodeling decreased in the male mice, a different phenotype emerged. Osteoclast formation became elevated in the male mice, leading to a low trabecular bone mass phenotype. In contrast, the female *Dmp1Cre.Socs3*^*f/f*^ mice maintained a normal level of bone resorption, and continued to accrue trabecular bone owing to a higher level of bone formation^87^.

These phenotypes were also unique to the appendicular skeleton, where the high bone mass was abnormal in its organization. The usual distinction between the thickened, dense cortical shell of bone and the light network of trabecular bone was obscured^87^. Normally, cortical bone develops at the growing metaphysis through a process of trabecular consolidation^88^. This process was delayed in the *Dmp1Cre.Socs3*^*f/f*^ mice, particularly in females, and the same was observed in gonadectomized male *Dmp1Cre.Socs3*^*f/f*^ mice treated with estradiol. These results indicate that the process by which cortical bone develops requires SOCS3-negative feedback, specifically in osteocytes. In this work, it was not possible to determine the particular osteocyte-specific cytokine critical for these outcomes. In fact, IL-6 deletion had no effect on the female mice, but the phenotype in the males intensified, becoming equivalent to that acquired by the females^87^.

Immature, highly porous, and poorly defined cortical bone was also observed in mice with SOCS3 deletion targeted to osteoblast and chondrocyte progenitors using *Col2.Cre*^89^, confirming the phenotype of the osteocyte-specific knockout mice^87^. In addition, *Col2Cre-*targeted SOCS3 deletion led to a 10% reduction in bone length, with altered proportions of proliferating and hypertrophic chondrocytes. Earlier studies in primary chondrocyte cultures from the same mice showed elevated STAT1, STAT3, and STAT5 signaling resulted from SOCS3 deletion^90^. However, the growth plate chondrocytes of *Col2Cre.Socs3*^*f/f*^ mice did not exhibit increased STAT3 phosphorylation. Instead, they showed increased MAPK phosphorylation^89^. In contrast to the *gp130*^*ΔSTAT/ΔSTAT*^ mice, which have systemically increased downstream MAPK signaling, there was no evidence of early growth plate closure detected in the *Col2Cre-*targeted SOCS3-deficient animals. Clearly multiple intrinsic factors regulate bone lengthening through STAT3 and MAPK signaling in chondrocytes.

More recently, it has been confirmed that osteocytes in *Dmp1Cre.Socs3*^*f/f*^ mice show prolonged STAT3 phosphorylation, both endogenously, and in response to OSM, LIF, and IL-11^91^. This suggests that OSM, LIF, and IL-11 are the SOCS3-dependent cytokines that must be suppressed for cortical bone to form and mature appropriately. When *Dmp1Cre.Socs3*^*f/f*^ mice were crossed with mice lacking gp130 in the same targeted cells, i.e., *Il6st-flox* (gp130) mice, the delayed cortical bone development was rescued. Further analysis confirmed that this outcome was owing to reduced numbers of osteoclastic resorptive foci within cortical bone and reduced STAT3 phosphorylation in the osteocytes^91^. These findings indicate that gp130-dependent STAT3 signaling in osteocytes must be suppressed to limit osteoclastic resorption and enable consolidation of cortical bone. The highly localized nature of this phenotype remains a topic of interest; osteoclast formation is not elevated in trabecular bone, suggesting that cytokine expression in the developing cortical structure may control the segregation of bone into cortical and trabecular components, perhaps in response to mechanical loads experienced during normal movement.

## Summary

**Fig. 2:**
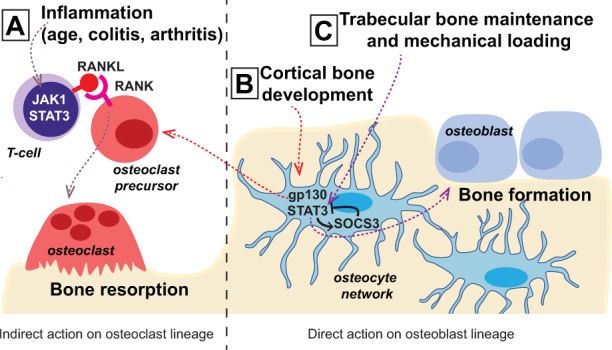
Context-dependent cytokine actions through gp130, JAK1, STAT3, and SOCS3 in bone formation and resorption. **a** Inflammation during colitis, age-related bone loss, and rheumatoid arthritis leads to increased RANKL production by T cells or related inflammatory cells at sites of inflammation through JAK1 and STAT3. This indirect action increases osteoclast formation on the bone surface. **b** During cortical bone development, osteocytes respond to IL-6 family cytokines in the local environment, induce STAT3 signaling, and depend on SOCS3-negative feedback to prevent excessive osteoclast formation. **c** In the trabecular bone network, normal physiological production of IL-6 family cytokines promotes bone formation and requires feedback from SOCS3. Mechanical loading also induces bone formation through STAT3 signaling in osteocytes, but whether this is induction is dependent on gp130 or SOCS3-negative feedback is not yet known.

In conclusion, normal bone structure and strength are dependent on JAK1/STAT3/SOCS3 signaling (Fig. 2). In general, osteoclast formation is stimulated indirectly by the IL-6 family of cytokines through these pathways via actions that promote RANKL expression in supportive cells. This is particularly relevant under conditions of systemic or local inflammation, where JAK1/STAT3 activation in T cells and in osteoblasts provides RANKL to osteoclast precursors to promote their differentiation. G-CSF also modifies both bone formation and resorption, but both of its actions appear to be indirect. In bone remodeling, when bone resorption is promoted through gp130-STAT3, bone formation is stimulated through an IL-6-dependent coupling mechanism. STAT3 activity in osteocytes is also critical for normal levels of bone formation, including bone formation in response to mechanical loading; when this pathway is hyperactivated in osteocytes by the deletion of SOCS3, excessive new bone is formed, and the normal formation of the thick cortex of bone is delayed owing to the hyperactivity of gp130-STAT3 signaling.
